# Adverse childhood experiences affect health outcomes for adults in North Dakota: 2019–2022 BRFSS population profile

**DOI:** 10.3389/fpubh.2025.1517431

**Published:** 2025-05-30

**Authors:** Ramona A. Danielson, Matthew Schmidt, Miranda A. Griechen

**Affiliations:** ^1^Department of Public Health, North Dakota State University, Fargo, ND, United States; ^2^Special Projects and Analytics Unit, Health Statistics and Performance Section, North Dakota Department of Health and Human Services, Bismarck, ND, United States

**Keywords:** adverse childhood experiences, health conditions, health behaviors, behavioral risk factor surveillance system, trauma-informed approaches

## Abstract

**Introduction:**

Adverse childhood experiences (ACEs) have been linked to chronic health conditions, at-risk behaviors, and reduced quality and length of life. Public health interventions targeting childhood trauma require an investigation of overall prevalence of ACEs and associations with outcomes and behaviors.

**Methods:**

We created an ACE score using aggregated North Dakota (ND) Behavioral Risk Factor Surveillance System (BRFSS) data from 2019 to 2022. Adjusted odds ratios for selected chronic conditions, health risk behaviors, and health burdens were obtained using a logistic regression model.

**Results:**

ND adults with 4+ ACEs had more than a 2x increased risk for chronic kidney disease, cardiovascular problems, asthma, arthritis, currently smoking, and frequent poor physical health and more than a 4x increased risk for COPD, using marijuana, frequent poor mental health, and having a depressive disorder. A 2-fold increased risk for frequent poor physical, frequent poor mental health, and having a depressive disorder was seen for ND adults with 1–3 ACEs.

**Discussion:**

ACEs, especially for ND adults with an ACE score of 4+, are associated with poorer mental and physical health outcomes. Using marijuana had the strongest association with health risk behaviors and having a depressive disorder had the strongest association of the health conditions in the study. Our results emphasize the importance of evidence-based ACE prevention strategies and trauma-informed approaches that public health officials and policy makers in ND and across the nation can use to help build resilience, prevent ACEs, and improve well-being across the population.

## Introduction

Health is multifaceted, and many factors play a role in the acquisition of health and life expectancy. Personal responsibility plays a role in health and behavior; however, the environments in which people live, learn, and work affect their quality of life and well-being (1). Since Felitti et al. (2) began studying adverse childhood experiences (ACEs) in the mid-1990s, investigation and analyses of the impacts of ACEs have become widespread. ACEs are traumatic experiences that occurred before age 18, and frequently are measured as factors of abuse and neglect (physical, emotional, sexual) as well as dynamics in the household (e.g., divorce, an incarcerated parent) (2, 3). Individual ACEs measure stresses that a child experiences, and tallying an ACE score based on how many ACEs a person experienced before age 18 becomes a measure of the “dose” of toxic stress a child experiences that can influence physical and cognitive development (4). While the adaptations that the child’s developing body makes may be adaptive to the threatening environment at the time, studies have shown that cumulative effects are harmful over time, especially in the absence of buffering factors (4, 5).

Individuals who have accumulated higher ACE scores (e.g., 4+ ACEs) have higher prevalence of risky behaviors and chronic conditions, and have a shorter life expectancy compared to people with 0 ACEs [e.g., (2, 6)]. Among adults with high cumulative ACEs, extant literature has found an increased likelihood of being smokers (7), users of marijuana (8), and heavy/binge drinkers (9–11). Individuals with higher ACEs are also less likely to engage in some protective behaviors, like cancer screening (12). Individuals with higher ACE scores have a higher risk for chronic diseases, including poor cardiovascular health (13–15), diabetes (16, 17), obesity (13), chronic pulmonary obstructive disease (COPD) (13), asthma (18), and higher symptom severity for systemic lupus erythematosus (19). Higher ACE scores are also associated with increased prevalence of depression and other mental health conditions (20, 21).

In its 2024–2029 State Health Improvement Plan (SHIP), the North Dakota Department of Health and Human Services (NDHHS) calls for the use of data to identify priorities for communities and develop strategic initiatives to adapt and meet the health needs of North Dakotans (45). Furthermore, a collective commitment has been made to strengthen childhood opportunities and reduce the prevalence of ACEs. A better understanding of ACEs provides an opportunity to analyze behaviors and conditions that may be associated with lifestyle choices, chronic health conditions, life expectancy, enjoyment of personal liberties, and potential health care burden among adults in North Dakota (ND). For these reasons, ND has chosen to include an optional module about ACEs in its Behavioral Risk Factor and Surveillance System (BRFSS) on a recurring basis (3).

This paper examines the landscape of ACEs in the ND adult, noninstitutionalized population. In addition to understanding the prevalence of ACEs among different demographic groups, this paper explores the association of higher cumulative ACEs with critical population health dimensions (e.g., chronic conditions, health burdens, and health risk behaviors). Our analysis is the first population profile for ACEs and analysis of their impacts of health outcomes in ND. Public health officials and other stakeholders in ND can use these findings to inform interventions. Additionally, while there are aspects of ND that may limit generalizability to other jurisdictions, this paper contributes to the body of knowledge exploring the impacts of ACEs across the lifespan.

## Methods

### Ethical considerations

This study analyzed data from the ND Behavioral Risk Factor Surveillance System (BRFSS), which is an annual, statewide population-based telephone survey conducted by the NDHHS in collaboration with the CDC (44). The CDC determined that the BRFSS information collection is exempt from requirements of Institutional Board Review in 45 CFR 46 (42). NDHHS determined the study did not need Institutional Review Board review because the study utilized secondary data. No identifying information was included in the dataset provided for data analysis. Data were stored in password-protected computers accessed only by the analysts, and all results are reported in aggregated form.

### Data source

This analysis used aggregated ND BRFSS data from 2019 to 2022. ND BRFSS collects information on health behaviors and chronic disease among noninstitutionalized adults aged 18 years or older. During the study period, 20,065 respondents participated in the ND BRFSS including 5,534 in 2019, 4,469 in 2020, 5,909 in 2021, and 4,153 in 2022. The calculated response rates follow standards set by the American Association for Public Opinion Research—60.8% in 2019, 55.6% in 2020, 53.5% in 2021, and 58.4% in 2022. While response rates during the COVID pandemic (2020, 2021, and 2022) were lower than pre-pandemic (2019), the response rates met the requested standard. Of the total survey participants, 2,021 (10.1% of the total sample) had missing data for the entire ACEs optional module, producing an analytical sample for this study of 18,044.

### ACEs variables

The four-year study period comprises survey years in which ND chose to include the ACEs optional module. Although the 2021 and 2022 ACEs module included two additional questions (i.e., emotional neglect, physical neglect), we excluded these questions due to lack of data across our study period. Thus, this analysis focused on 8 ACE domains regarding respondents’ experiences prior to turning 18 (Table 1). These 8 domains captured child abuse (e.g., physical abuse, emotional abuse, sexual abuse) and household dynamics (e.g., living with a depressed or mentally ill caregiver, an alcoholic or an adult who used illegal drugs, or a caregiver who had been incarcerated; having parents who were divorced or separated; or intimate partner violence in the home).

**Table 1:** ND BRFSS survey operationalization and prevalence of eight adverse childhood experiences (ACEs) among North Dakota adults, 2019–2022.

**ACE Category**	**Experiences Before Age 18:**	**% (95% CI)**
**ABUSE AND NEGLECT**		
**1**	**Emotional Abuse**	**35.9 (34.9 – 36.9)**
	Did a parent or adult in your home **ever** swear at you, insult you, or put you down?	
**2**	**Physical Abuse**	**22.2 (21.3 – 23.0)**
	Not including spanking, did a parent or adult in your home **ever** hit, beat, kick, or physically hurt you in any way?	
**3**	**Sexual Abuse**	**11.3 (10.6 – 12.0)**
	Did anyone at least 5 years older than you or an adult **ever** touch you sexually? Yes and/or	10.1 (9.4**–** 10.7)
	Did anyone at least 5 years older than you or an adult **ever** try to make you touch them sexually? Yes and/or	7.3 (6.7 – 7.9)
	Did anyone at least 5 years older than you or an adult **ever** force you to have sex? Yes	4.7 (4.2**–** 5.2)
**HOUSEHOLD DYNAMICS**		
**4**	**Substance Abuse in the Home**	**28.2 (27.3 – 29.1)**
	Did you live with anyone who was a problem drinker or alcoholic? Yes and/or	24.7 (23.8**–** 25.6)
	Did you live with anyone who used illegal street drugs or who abused prescription medications? Yes	10.7 (10.0**–** 11.4)
**5**	**Parental Separation or Divorce** Were your parents separated or divorced? Yes	**23.9 (22.9 – 24.8)**
**6**	**Household Member with Mental Illness** Did you live with anyone who was depressed, mentally ill, or suicidal? Yes	**19.6 (18.7 – 20.5)**
**7**	**Intimate Partner Violence** Did parents or adults in the household **ever** slap, hit, kick, or beat each other up? Yes	**14.7 (14.0 – 15.5)**
**8**	**Incarcerated Household Member** Did you live with anyone who served time or was sentenced to serve time in a prison, jail, or other correctional facility? Yes	**8.7 (8.0 – 9.3)**

CI, Confidence interval. Bolded text represents overarching ACE category and its respective % (95% CI).

### Outcome variables

We explored outcomes related to chronic conditions, health burdens, and health risk behaviors. Chronic conditions included if they had ever been told they have diabetes; asthma; cardiovascular disease (heart attack/myocardial infarction, angina/chronic heart disease, or stroke), arthritis (some form of arthritis, rheumatoid arthritis, gout, lupus, or fibromyalgia), any cancer (skin cancer, melanoma, or any other types of cancer), kidney disease, chronic obstructive pulmonary disease (COPD; including emphysema or chronic bronchitis), or a depressive disorder (including depression, major depression, dysthymia, or minor depression). We also included obesity (having a body mass index >30). The health burden measures included frequent poor physical health (physical health was not good for 14+ of the past 30 days for reasons including physical illness or injury) and frequent poor mental health (mental health was not good for 14+ of the past 30 days for reasons including stress, depression, and problems with emotions). Health risk behaviors included current smoker (some days or every day), current marijuana user (used marijuana at least 1 day in the past 30 days), and binge drinker (male respondents who drank 5+ drinks on one occasion/female respondents who drank 4+ drinks on one occasion).

### Statistical analysis

Survey design effects were applied, including stratification and weighting. To account for multiple survey years, uniform weights were created by dividing final respondent weights by the number of survey years. ACE scores were assigned by coding affirmative responses to 8 ACE domains; thus, each respondent had an ACE score ranging from 0 to 8. Cronbach’s alpha was calculated to determine the reliability of the ACE index (22); alpha was 0.74, indicating an acceptable level of internal consistency. The ACE score was recoded for analysis into 3 categories: 0 ACEs (no exposure), 1–3 ACEs (low to moderate exposure), and 4+ ACEs (high exposure). A cutoff score of 4+ is the level at which extant research has consistently shown association with more negative outcomes (23). Multiple logistic regression assessed the independent relationship between ACEs groups and selected outcomes, while accounting for relevant confounders including respondent sex, race/ethnicity, age, and education. We did not adjust for annual household income because the variable does not consider household size. Statistical significance was determined using Chi-square test of independence. All analyses were conducted in SAS version 9.4 (24).

## Results

Half of the study’s population was male (50.6%; see Table 2). More than 4 in 5 ND adults were White, non-Hispanic (85.8%); the next largest population was American Indian/Alaska Native, non-Hispanic at 4.2%. Nearly half of the population was ages 18–44 (47.6%); more than 1 in 5 ND adults were 65+ (22.8%). A small proportion of the population had not graduated high school (7.2%), 26.0% were high school graduates, 38.9% had some college, and 28.0% were college graduates. More than 3 in 5 ND adults had incomes of at least $50,000 (62.1%); 14.2% had incomes of less than $25,000.

**Table 2:** Overall prevalence of sociodemographic characteristics of North Dakota adults and prevalence by adverse childhood experiences (ACE) score, 2019–2022.

	Overall prevalence % (95% CI)	Prevalence by ACE Score			
		0 ACEs	1–3 ACEs	4+ ACEs	Total %
		% (95% CI)	% (95% CI)	% (95% CI)	
Total Population	NA	39.0 (38.1–40.0)	44.1 (43.1–45.0)	16.9 (16.1–17.7)	
Sex*					
Male	50.6 (49.6–51.6)	40.3 (39.0–41.6)	44.8 (43.5–46.2)	14.9 (13.9–15.9)	100.0
Female	49.4 (48.4–50.4)	37.8 (36.4–39.1)	43.2 (41.8–44.7)	19.0 (17.8–20.3)	100.0
Total	100.0				
Race/Ethnicity*					
White, NH	85.8 (85.0–86.7)	41.1 (40.1–42.1)	44.0 (42.9–45.0)	15.0 (14.1–15.8)	100.0
Black, NH	2.8 (2.4–3.3)	41.3 (33.2–49.4)	41.7 (33.8–50.0)	17.0 (11.1–22.9)	100.0
AI/AN, NH	4.2 (3.8–4.6)	20.1 (16.4–23.9)	39.9 (34.9–44.8)	40.0 (34.9–45.1)	100.0
Other, NH	3.8 (3.3–4.2)	27.9 (22.4–33.5)	46.3 (39.8–52.7)	25.8 (20.1–31.5)	100.0
Hispanic	3.4 (2.9–3.9)	22.0 (16.0–28.0)	49.4 (42.0–56.8)	28.6 (22.1–35.2)	100.0
Total	100.0				
Age Group*					
18–24	13.2 (12.4–14.1)	29.2 (25.9–32.5)	45.4 (41.8–49.1)	25.3 (22.2–28.5)	100.0
25–34	18.2 (17.3–19.1)	27. 8 (25.3–30.2)	47.0 (44.2–49.9)	25.2 (22.6–27.7)	100.0
35–44	16.2 (15.4–17.0)	34.9 (32.4–37.4)	42.2 (39.6–44.8)	22.9 (20.6–25.2)	100.0
45–54	13.3 (12.7–14.0)	37.4 (35.0–39.8)	47.3 (44.8–49.8)	15.3 (13.5–17.0)	100.0
55–64	16.2 (15.6–16.8)	43.2 (41.3–45.2)	44.2 (42.2–46.1)	12.6 (11.2–14.0)	100.0
65+	22.8 (22.2–23.4)	54.6 (53.3–56.0)	40.2 (38.8–41.5)	5.2 (4.6–5.8)	100.0
Total	100.0				
Education*					
Less than H.S.	7.2 (6.5–7.8)	29.9 (25.6–34.1)	44.2 (39.1–49.2)	26.0 (21.4–30.5)	100.0
H.S. Graduate	26.0 (25.1–26.9)	36.2 (34.5–38.0)	43.9 (42.0–45.8)	19.9 (18.2–21.6)	100.0
Some College	38.9 (37.9–39.8)	39.2 (37.6–40.8)	43.5 (41.9–45.2)	17.3 (15.9–18.7)	100.0
College Graduate	28.0 (27.2–28.8)	43.7 (42.2–45.2)	45.0 (43.5–46.5)	11.3 (10.3–12.3)	100.0
Total	100.0				
Annual Household Income*					
<$25,000	14.2 (13.4–15.0)	31.5 (28.8–43.1)	41.9 (38.9–44.9)	26.6 (23.8–29.5)	100.0
$25,000–$49,999	23.7 (22.8–24.6)	36.6 (34.6–38.6)	45.5 (43.3–47.7)	17.9 (16.0–19.9)	100.0
$50,000+	62.1 (61.0–63.1)	39.9 (38.6–41.2)	44.8 (43.4–46.1)	15.3 (14.2–16.4)	100.0
Total	100.0				

CI, Confidence interval; NH, non-Hispanic; AI/AN, American Indian/Alaska Native; H.S., High school; *p < 0.01; *p*-values obtained by Chi-square test for independence.

Among our selected outcomes, obesity was the most prevalent chronic condition (35.2%), followed by arthritis (28.1%; see Table 3). One in five respondents indicated they had a depressive disorder (19.6%). More than 1 in 10 respondents indicated they had asthma (13.0%) and had any cancer (11.7%). One in 10 respondents indicated they had diabetes (9.8%). A larger proportion of respondents indicated they had frequent poor mental health (12.1%) than frequent poor physical health (9.4%). One in five respondents said they were a binge drinker (21%), 15.5% said they were a current smoker, and 8.3% said they currently used marijuana.

**Table 3:** Prevalence of selected outcomes and associations between adverse childhood experiences (ACE) score and selected outcomes for North Dakota adults, 2019–2022: multivariable regression model.

Outcomes^1^	Overall prevalence % (95% CI)	Comparison of ACE score to 0 ACEs	
		1–3 ACEs vs. 0 ACEs AOR (95% CI)	4+ ACEs vs. 0 ACEs AOR (95% CI)
Chronic Conditions			
Diabetes	9.8 (9.3–10.3)	1.3 (1.2–1.5)^	1.5 (1.2–1.8)^
Asthma	13.0 (12.3–13.7)	1.5 (1.3–1.7)^	2.8 (2.3–3.3)^
Cardiovascular disease	8.3 (7.8–8.7)	1.4 (1.2–1.6)^	2.6 (2.1–3.3)^
Arthritis	28.1 (26.5–29.8)	1.5 (1.2–1.8)^	2.9 (2.2–3.9)^
Any cancer	11.7 (11.2–12.2)	1.2 (1.1–1.3)^	1.6 (1.3–2.0)^
Kidney disease	3.1 (2.8–3.4)	1.5 (1.2–1.8)^	2.2 (1.6–3.0)^
COPD	5.4 (5.0–5.8)	1.8 (1.5–2.1)^	4.4 (3.4–5.7)^
Depressive disorder	19.6 (18.7–20.4)	2.7 (2.4–3.1)^	6.8 (5.8–8.0)^
Obese (BMI > 30)	35.2 (34.2–36.2)	1.3 (1.2–1.4)^	1.6 (1.4–1.8)^
Health Burdens			
Frequent poor physical health	9.4 (8.9–10.0)	2.0 (1.7–2.2)^	3.7 (3.0–4.5)^
Frequent poor mental health	12.1 (11.4–12.8)	2.5 (2.1–3.0)^	5.9 (4.8–7.3)^
Health Risk Behaviors			
Current smoker	15.5 (14.8–16.3)	1.5 (1.3–1.7)^	3.1 (2.6–3.7)^
Current marijuana use	8.3 (7.8–9.0)	2.0 (1.6–2.5)^	4.7 (3.7–6.0)^
Binge drinker	21.0 (20.0–22.0)	1.2 (1.1–1.4)^	1.4 (1.1–1.7)^

Estimates adjusted (controlled) for sex, race/ethnicity, age, education; AOR, Adjusted odds ratio; CI, Confidence interval; COPD, chronic obstructive pulmonary disease; BMI, body mass index; ^*p*-value < 0.05.

1 See Methods section for descriptions of outcomes.

Table 1 shows the eight ACEs questions asked in the survey, with prevalence. Emotional abuse was the most common form of abuse experienced before age 18 reported by ND adults (35.9%), followed by physical abuse (22.2%) and then sexual abuse (11.3%). For household dynamics experienced before age 18, substance abuse in the home was most common (28.2%), followed by parental separation or divorce (23.9%), a household member with mental illness (19.6%), intimate partner violence (14.7%), and living with a household member who had been incarcerated (8.7%).

As shown in Table 2, 39.0% of ND adults indicated they had not experienced any ACEs, 44.1% had experienced 1–3 ACEs (considered a low to moderate ACE score), and 16.9% had experienced 4+ ACEs. The prevalence of high ACE scores differed significantly based on several demographic characteristics. A higher proportion of females had an ACE score of 4+ (19.0%) compared to males (14.9%). A higher proportion of American Indian/Alaska Native, non-Hispanic people (40.0%), Hispanic people (28.6%), and non-Hispanic people of other races (25.8%) had a high ACE score compared to White, non-Hispanic (15.0%) and Black, non-Hispanic people (17.0%). People with annual household incomes less than $25,000 (26.6%) were more likely to have an ACE score of 4+ compared to people of higher incomes (17.9% for $25,000–$49,999 and 15.3% for $50,000+). The proportions of high ACE scores decreased significantly with age, from those with younger ages (25.3% of those 18–24, 25.2% of those 25–34, and 22.9% of those 35–44), to people of middle age (15.3% of those 45–54 and 12.6% of those 55–64), to people of older age (5.2% of those 65+). The proportions of high ACE scores also decreased significantly as education increased. Among those with less than a high school degree, 26.0% had an ACE score of 4+, followed by 19.9% among those with a high school diploma, 17.3% with some college, and 11.3% among college graduates.

The results of the multiple logistic regression model showed significant results based on the level of exposure to ACEs (for low to moderate exposure as well as high exposure) compared to the reference group of people with no exposure to ACEs, after controlling for the effects of sex, race/ethnicity, age, and education (see Table 3).

Among chronic conditions, the adjusted odds ratios (AOR) showed a greater likelihood among people with ACEs compared to those without ACEs, especially among people with high ACE scores (4+ ACEs; see Table 3). The prevalence of depressive disorder was 6.8x higher among people with high ACE scores compared to people with ACE scores of 0 (2.7x for ACE scores of 1–3 compared to 0 ACEs). COPD was 4.4x more likely among people with high ACE scores (1.8x for 1–3), arthritis was 2.9x more likely (1.5x for 1–3), asthma was 2.8x more likely (1.5x for 1–3), cardiovascular disease was 2.6x more likely (1.4x for 1–3), and kidney disease was 2.2x more likely (1.5x for 1–3). The prevalence of any cancer was 1.6x more likely for people with high ACE scores (1.2x for 1–3), obesity was 1.6x more likely (1.3x for 1–3), and diabetes was 1.5x more likely (1.3x for 1–3).

When looking at the association of ACEs with health burdens, poor mental health was 5.9x more likely among people with ACE scores of 4+ (2.5x for 1–3), while frequent poor physical health was 3.7x more likely (2.0x for 1–3). Among health risk behaviors, people with a high ACE score were 4.7x more likely to be current marijuana users (2.0x for 1–3), 3.1x more likely to be current smokers (1.5x for 1–3), and 1.4x more likely to be binge drinkers (1.2x for 1–3).

## Discussion

BRFSS data reported for ND for 2011–2020 (25) provide an opportunity to look at ACEs over time; prevalence rates have remained fairly stable for ND adults. When compared to national BRFSS data and accounting for point estimates with their confidence intervals (25), our study showed a lower prevalence of sexual abuse in ND compared to the national prevalence (11.3% for ND vs. 12.6% nationally), parental separation or divorce (23.9% for ND vs. 28.4% nationally), and intimate partner violence (14.7% in ND vs. 17.2% nationally). ND adults reported a higher prevalence of household substance abuse (28.2% for ND vs. 26.5% nationally) and household mental illness (19.6% for ND vs. 17.3% nationally). The proportion of ND adults who reported 0 ACEs in this study was higher than the national proportion (39.0% for ND vs. 36.1% nationally), but consistent regarding scores of 4+ (16.9% for ND and 17.3% nationally).

Among ND adults in our study, 4+ ACEs was significantly more common among females, AI/AN people, younger ages (18–44), lower education levels (especially less than high school graduates), and lower annual household incomes (less than $25,000). The demographic patterns among ND adults were consistent with national patterns showing differences by sex, race, age, education, and income (25, 26). However, the proportion of adults who reported 4+ ACEs was slightly lower in ND compared to the nation overall among people ages 45–54 (15.3 and 18.6%, respectively) and ages 65+ (5.2 and 7.7%, respectively) (25). The proportion of Hispanics with 4+ ACEs was higher in ND than for the nation overall (28.6 and 18.6%, respectively). The proportion of AI/AN, non-Hispanic with 4+ ACEs in ND is comparable to the rate for AI/AN in the national data (40.0 and 32.4% respectively), after taking confidence intervals into consideration. The substantial rates of high ACEs among AI/AN people are a national trend (26), reflecting legacies of historical and intergenerational trauma (27).

As a cross-sectional study, BRFSS data reveal associations, rather than causation. However, the results of our study show consistent relationships between the level of exposure to ACEs and increased odds of chronic conditions and health burdens. In our study, the odds of having negative health outcomes were significantly higher for people who experienced ACEs compared to people who did not for all of the outcomes studied, with low to moderate ACE exposure and with high ACEs exposure; odds ratios for high ACE scores were consistently larger than for ACE scores of 1–3. Our results are consistent with studies showing a higher prevalence of chronic diseases with higher ACE scores, and a dose–response relationship for health conditions and behaviors associated with increasing ACE scores [e.g., (2, 28)]. When considering point estimates with their confidence intervals, analysis of Iowa BRFSS data (28) showed very similar patterns to ND BRFSS for various chronic conditions, with significantly higher odds ratios for diabetes, heart disease, cancer, COPD, and obesity. At 35.1%, ND was among 23 states with adult obesity rates over 35% in 2023 (29).

The association between ACEs and depression was even stronger in our study compared to the Iowa study. Along with substance abuse, mental health has frequently been identified as a top need in Critical Access Hospital Community Health Needs Assessments conducted in the state, especially in rural areas (30). The risk for mental health-related issues is of particular concern considering 46 of ND’s 53 counties were designated as mental health provider shortage areas in 2024 (31). In ND, there was only 1 mental health provider for every 420 residents in 2024; the national average was 1 for every 300 residents (32).

Extant research echoes our study results for smoking, marijuana use, and binge drinking. People with ACEs are more likely to be current smokers than those with 0 ACEs (7, 28). Increasing ACE scores have also been associated with a significant increase in marijuana use (8). For alcohol consumption behaviors, higher ACEs have shown an association with heavy drinking and binge drinking behavior (9, 10, 28, 33). Heavy alcohol consumption is a persistent challenge in ND. According to America’s Health Rankings (AHR), ND has the highest prevalence of heavy drinking and binge drinking in America at 22.3% in 2023 compared to 16.7% nationally (41).

## Limitations

There are important limitations to our study. ACEs are sensitive questions, especially concerning physical, emotional, or sexual abuse. As a result, the ND BRFSS ACEs module has a higher refusal rate for these questions than for other questions. The ACEs module was placed at the end of the questionnaire in order to minimize other missing data by respondents terminating the survey. Missing rates for these questions per survey are approximately 2–5% a survey year (resulting in 10.1% of our aggregate dataset). However, we retained survey responses in our analysis even if they had missing data if the cumulative ACE score from known items was at least 4, since we aggregated all scores of 4–8 ACEs into a cumulative category of 4+.

Selection bias could be a factor in our study in two ways. One, people who chose to not respond to the ACEs questions may have higher ACE scores. Two, people with higher ACE scores die at younger ages than people with lower or no ACEs exposure. This means that people with higher ACE scores are less likely to live to older ages (34), which may contribute to the lower percentage of high ACE scores we found among respondents ages 65+ in our study.

Recall bias is always a potential limitation for retrospective data. When doing surveillance on ACES, researchers typically do not ask for the exact age and time ACEs took place; not asking for these specifics is a way to reduce recall bias. While some researchers argue about the efficacy of a cumulative ACE score because it gives equal weight to each ACE (35), the general consensus among researchers is that this specificity is not needed because ACEs tend to co-occur and the cumulative effect of the ACE score, i.e., the “dose” of toxic stress, is seen as the mechanism impacting health outcomes (4).

Social desirability bias may also affect the results of the study. Participants may feel shame revealing certain lifestyle choices or feel shame surrounding mental health conditions or ACEs. Therefore, they may provide a response that is more socially acceptable than their truthful answer. Hence, our study may be an undercount of all individuals with ACEs.

Aggregating the ND BRFSS data across 4 years of data collection allowed for more in-depth analyses. However, there were limitations resulting from aggregating our data. Although sexual minorities often have higher ACE scores and mental health conditions than their peers (36), we were not able to include a variable about sexual orientation in our analyses. ND BRFSS added questions regarding sexual orientation beginning with the 2021 survey, but the variable was not available for our full study period. Additionally, while the CDC optional ACEs module now collects information about two additional ACEs (emotional neglect and physical neglect), we did not have these variables across our full study period and excluded them from our analyses.

Furthermore, our aggregated time period includes data from before the COVID pandemic and during the COVID pandemic. Symptoms of anxiety and depression increased dramatically among adults during the COVID pandemic (37, 38, 46). Younger adults (ages 18–29) and women—especially mothers—were more likely to report mental health-related impacts than other groups (38). Research by Fortier et al. (37) found that self-rated physical and mental health decreased with every COVID-related stressor adults had experienced in the past month. Increased odds of “fair or poor” self-rated physical or mental health were associated with stressors like feeling lonely or isolated, needing to access health care (for any reason), and uncertainty about the future. Research by Doom et al. (46) with college students found that high ACEs were associated with more anxiety and greater depression during the COVID pandemic, and that college students with higher ACE scores experienced significantly more COVID-related stressors. Thus, the dynamic of COVID could contribute to even stronger associations between high ACE scores and prevalence of the outcomes we studied than would perhaps be found prior to or since the pandemic. A strength of our study in the context of the influence of COVID-related experiences on health and well-being is that our analyses focused on the associations with ACEs in an aggregated time period, rather than the extent to which health outcomes have changed over time. As ND BRFSS continues surveillance of ACEs on a recurring schedule, we can compare post-pandemic results to the results of this study.

## Conclusion

The results of our study show consistent relationships between the level of exposure to ACEs and increased odds among the outcomes we examined, pointing to an important need for policy makers, health care providers, and public health professionals to consider ACEs when promoting well-being among ND residents. Influencing policy makers to support future ACEs legislation has been a challenge in many jurisdictions, however (39). Providing educational materials, sharing knowledge surrounding ACEs, and discussing the importance of trauma-informed approaches to service delivery and community development may influence the decision making of residents, health professionals, and policy makers (40). Stakeholders can utilize guidance such as the CDC’s six strategies for prevention of ACEs, which emphasize resilience, optimism, and opportunities for preventing and helping individuals with ACEs to prioritize prevention approaches (43). The strategies include strengthening economic supports for families, supporting social norms that protect against violence and adversity, ensuring a strong start to development for children, enhancing skills to help parents with daily challenges, connecting youth to caring adults and activities, and intervening to lessen the effects of ACEs. These evidence-based strategies can be used to prevent ACEs from occurring as well as limit the accumulation of higher ACE scores for children. For adults, these strategies can help mitigate the effects of ACEs and promote greater well-being.

## Data Availability

Publicly available datasets were analyzed in this study. This data can be found at: https://www.cdc.gov/brfss/annual_data/annual_data.htm.
